# A Multimodal Physical Program Combining Abacus Use and Exercise to Improve Motor Coordination and Flexibility in Primary School Children

**DOI:** 10.3390/jfmk10030255

**Published:** 2025-07-05

**Authors:** María del Carmen Carcelén-Fraile, Agustín Aibar-Almazán, Alba Rusillo-Magdaleno, Alberto Ruiz-Ariza

**Affiliations:** 1 Department of Educational Sciences, Faculty of Social Sciences, University of Atlantico Medio, 35017 Las Palmas de Gran Canaria, Spain; 2 Department of Health Sciences, Faculty of Health Sciences, University of Jaen, 23071 Jaen, Spain; 3 Faculty of Educational Sciences, University of Jaen, 23071 Jaen, Spain; arusillo@ujaen.es (A.R.-M.); arariza@ujaen.es (A.R.-A.)

**Keywords:** physical exercise, abacus, flexibility, motor coordination, children

## Abstract

**Background**: During early childhood, physical development plays a critical role in overall growth, influencing not only health but also academic and social outcomes. In this context, the present randomized controlled trial aims to analyze the effects of a combined intervention using physical exercise and abacus training on flexibility and motor coordination in early primary school children. **Methods**: A total of 82 girls and boys participated in this study, of which 41 belonged to the experimental group that carried out a combined training of physical exercise and the use of an abacus for 12 weeks and 41 to a control group that did not receive any intervention. Flexibility was measured with the Sit and Reach test and motor coordination with the motor coordination test. **Results**: In this study, statistically significant improvements were observed in flexibility in both the right and left legs and in all domains related to motor coordination in the training group. **Conclusions**: The results of this study support the effectiveness of a multidimensional approach that integrates physical and cognitive activities, such as the use of the abacus, to improve coordination and flexibility in children, contributing to comprehensive development in early childhood education.

## 1. Introduction

Children in the first cycle of primary school go through a stage of accelerated development in which they consolidate essential skills in both the physical and cognitive fields [1,2]. Physical development during the first years of primary education is essential for the long-term health and well-being of children [3]. At this age, students go through a phase of accelerated growth in which they consolidate essential skills in both the physical and cognitive fields [4]. Their curiosity and energy, together with a great capacity for adaptation, promote an integral development that encompasses both academic learning and physical and social development [5]. This stage of high plasticity allows their gross and fine motor skills to be shaped and optimized through activities that stimulate balance, strength, endurance, and coordination [6]. In this sense, school environments represent a unique opportunity to promote physical development through approaches that integrate movement and academic learning in a synergistic way [7].

Physical development at this stage is not only associated with improved motor skills, but also with benefits that include bone strengthening, body weight regulation, and cardiovascular health, aspects that are essential for healthy growth [8,9]. Multiple studies have demonstrated that children who engage in regular physical activity are less likely to develop chronic illnesses later in life and tend to exhibit higher functional capacity in their everyday activities [10,11]. Likewise, it has been shown that they not only contribute to the development of strength and endurance, but also promote better motor integration, helping children reach important milestones in their motor development [12]. In addition, there is a close relationship between physical movement and the development of cognitive processes such as attention, inhibition, and working memory, components that play a key role in self-regulation and motor learning [13,14,15]. Therefore, it is essential to implement interventions that promote physical exercise and motor development, providing children with opportunities to enhance their physical and cognitive growth through structured movement-based experiences [16].

In this context, the use of the physical exercise and abacus emerge as complementary resources in the classroom that can help optimize the physical development of children [17,18]. Traditionally, the abacus has been used as a tool in mathematical learning, but recent research suggests that its use also encourages visual–motor and fine motor skills by involving rhythmic and precise movements that improve coordination between the eyes and the hands. Furthermore, it supports muscle development in the hands and fingers, increasing precision and strength, which is considered beneficial for a wide range of fine motor activities and overall motor proficiency in children [19,20,21]. Thus, the abacus becomes a resource that allows stimulating not only logic and concentration, but also fine physical skills, which adds a significant component to the child’s comprehensive development in the school context [22,23]. Along with the abacus, the inclusion of physical exercise such as active games or structured sessions contributes to the development of both gross and fine motor skills, both crucial for complete physical development [24], and physical exercises of resistance, strength and flexibility strengthen muscles and improve children’s coordination, balance, and cardiovascular health [25,26].

Among the various educational tools available to enhance cognitive development in children, the abacus stands out for its dual role as both a mathematical and neurodevelopmental resource. In recent years, interest in abacus-based mental calculation (AMC) has grown due to its potential to stimulate executive functions, including working memory, attention, and cognitive flexibility—key components for learning and motor regulation. Lee et al. (2022) conducted a meta-analysis confirming that abacus training leads to measurable improvements in working memory and mathematical reasoning in school-aged children [27]. Neuroimaging research by Tanaka et al. (2002) further demonstrated that children trained in abacus methods activate both prefrontal and parietal regions more efficiently during mental tasks, indicating enhanced neurocognitive processing [28].

These findings support the abacus not merely as a calculation aid, but as a tool that engages complex cognitive systems through multisensory stimulation, especially when integrated with motor actions such as finger manipulation. Rueda et al. argue that early training in attention and executive regulation fosters more efficient brain maturation and self-control [29]. As such, the abacus provides a structured, engaging, and physically interactive platform to reinforce both academic and developmental competencies.

Moreover, recent evidence by Escolano-Pérez and Herrero-Nivela) [30] confirms that the relationship between cognitive and motor development is not unidirectional, but bidirectional and interdependent. Their findings underscore the importance of integrating physical and cognitive stimuli in educational programs to promote holistic development during early childhood [30].

Despite these insights, current school practices often treat physical and cognitive learning as separate domains, missing the opportunity to address them in a unified and developmentally meaningful way. This separation is particularly problematic during the early years of primary education, a period marked by heightened neuroplasticity and rapid motor maturation. In this context, the lack of integrated interventions that simultaneously target executive function and motor coordination represents a critical gap in the educational field.

The present study responds to this need by proposing an innovative, classroom-based intervention that combines abacus-based cognitive tasks with structured physical exercises. This integrated approach is designed to enhance flexibility and motor coordination through cognitively enriched movement, offering a novel model for addressing the multifaceted needs of children in early schooling. By bridging the gap between cognitive and motor development, this study not only fills a void in current educational practice, but also contributes new evidence to support holistic pedagogical strategies. Therefore, the main objective of this study is to analyze the effects of a combined program of structured physical exercises and abacus training on flexibility and motor coordination in children in the lower cycle of primary education. It is hypothesized that children participating in this integrated intervention will show significant improvements in flexibility and different dimensions of motor coordination compared to a control group that did not receive this intervention.

## 2. Materials and Methods

### 2.1. Design Study

The present study utilizes a randomized controlled trial to examine how a program that merges abacus-based learning with physical exercise influences the development of physical of children in the initial phase of primary education. Prior to launching this study, legal guardians provided informed consent, and ethical clearance was granted by the Ethics Committee at the University of the Middle Atlantic (CEI/01–013).

### 2.2. Participants

**Figure 1:**
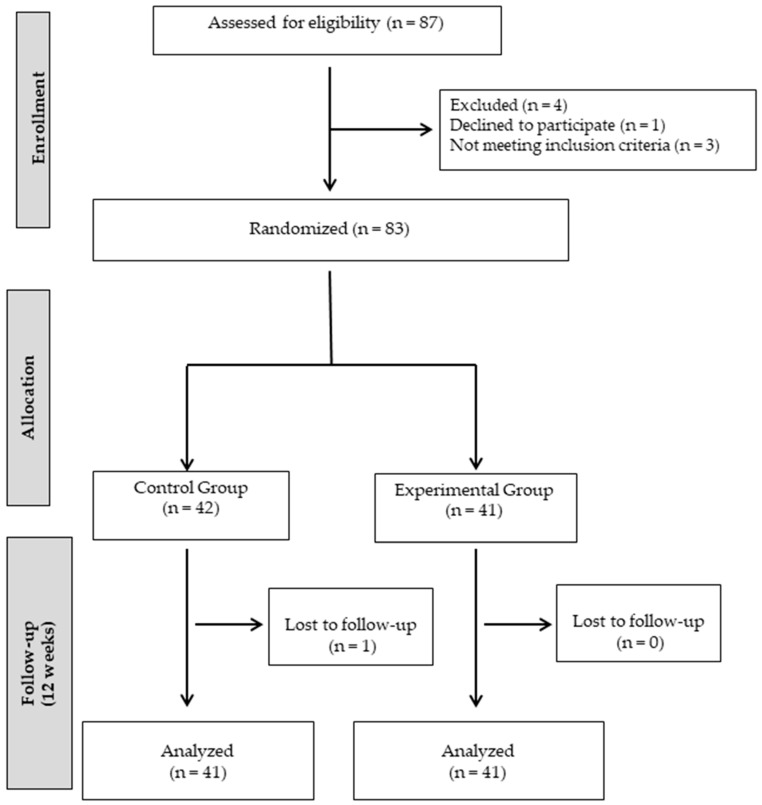
Participant flowchart for the study process.

A total of 87 children in the first cycle of primary education were initially approached for this study. Of these, 83 met the eligibility requirements and agreed to participate. The inclusion criteria included: (i) enrollment in the first cycle of primary education; (ii) capacity to participate in moderate-intensity physical exercise without medical limitations, along with prior familiarity with abacus usage; and (iii) willingness to participate voluntarily, with informed consent obtained from parents or legal guardians. Exclusion criteria were as follows: (i) the presence of medical conditions that contraindicate physical exercise or safe abacus use (e.g., cardiac conditions, significant respiratory issues, or motor impairments); (ii) severe cognitive or learning difficulties that could hinder effective engagement with the abacus or equitable participation in the intervention; and (iii) concurrent enrollment in other extracurricular or educational programs aimed at cognitive or physical development, to minimize confounding variables. Ultimately, 82 students took part in this study, comprising 48 boys (58.50%) and 34 girls (41.50%) (Figure 1).

### 2.3. Procedure

Participant recruitment was carried out in a primary school located in the Andalusia region. The recruitment process began with an informative session aimed at the students’ legal guardians, during which all relevant aspects of the research were clearly explained. During this meeting, guardians received a detailed information sheet along with the consent forms required for participation. Only children whose parents signed informed consent and who met the inclusion criteria (aged 6–8 years, enrolled in the school’s first cycle of primary education, without diagnosed neurological or musculoskeletal disorders) were eligible to participate. Once consent was obtained and eligibility criteria were verified, baseline data collection was initiated. This involved administering questionnaires to the children both before the start of the intervention and after its completion.

Participants were then randomly assigned to one of two groups using a computer-generated random number sequence. Randomization occurred only after baseline assessments were completed, ensuring that group allocation could not influence initial data collection. The allocation followed a 1:1 ratio and was managed by an independent researcher not involved in the recruitment, assessment, or implementation of the intervention. To guarantee allocation concealment and minimize selection bias, group assignments were placed inside sequentially numbered, opaque, sealed envelopes. These were stored securely and opened only after the participant’s inclusion was confirmed. Neither the children, their teachers, nor the research team conducting assessments were aware of the group assignments, ensuring partial blinding of both participants and evaluators. Outcome assessments were conducted by trained personnel blinded to group allocation.

The experimental group took part in a structured program that combined physical exercise with cognitive tasks using the abacus. These sessions were conducted in the school gym during the regular physical education period, specifically from 12:00 to 12:45 p.m. In contrast, the control group continued their standard physical education lessons without any alterations or supplementary activities.

The program was implemented by the students’ regular teachers, who received training over a two-week preparation period before the intervention began. This training, led by the research team, included practical workshops on the correct use of the abacus, guidelines for integrating abacus-based exercises with physical exercises, and classroom management strategies to ensure active participation. The researchers provided teachers with detailed session plans and step-by-step instructional guides. The intervention was carried out in the school’s indoor gymnasium, which was adapted to allow simultaneous use of the abacus and execution of physical motor games. The school provided tables and mats for abacus activities, and cones, balls, and other materials for motor coordination exercises.

Members of the research team were present during the sessions to ensure that all activities were carried out as planned and according to protocol. Attendance and participation were carefully tracked using observation and attendance logs. To ensure data quality, any child who attended fewer than 80% of the sessions was excluded from the final analysis, as consistent participation was deemed necessary to maintain the reliability and internal validity of this study’s outcomes. To ensure full integration of the program into the school setting, coordination meetings were held with school administration and teaching staff prior to and during the intervention. Parents were kept informed about the progress and content of the sessions through regular written updates. They were also instructed to avoid enrolling their children in any external programs that might influence physical or cognitive development during the study period.

### 2.4. Outcomes

An independent researcher, who had no involvement in group allocation or intervention delivery, was responsible for collecting the data at two time points: before the intervention began and after its completion. The data included sociodemographic and anthropometric variables such as age, gender, height, weight, waist and hip circumference, waist-to-hip ratio, and Body Mass Index (BMI). Height measurements were taken using an Asimed T201–T4 stadiometer (New Delhi, India), while body weight was recorded with a Tefal digital scale accurate to within 100 g and with a capacity of up to 130 kg. BMI was calculated by applying the standard formula: weight in kilograms divided by the square of height in meters.

#### 2.4.1. Flexibility

To measure flexibility, the Sit and Reach test [31,32] was performed. The subject stood with their legs extended and unflexed, keeping their heels together and their toes 10–15 cm apart, resting against the vertical measuring plate, and joined both hands. During the test, with their arms and hands fully extended, the participant slowly flexed their upper body forward, gently moving the vernier scale forward with their fingertips (without making sudden movements) until reaching its maximum. The position of the longitudinal plate in the plane of the stapes represented the 0 point, and the measurements were recorded in centimeters: backwards for a negative value and forwards for a positive value. If the value was negative, the symbol “−” was used. In the present sample, the internal consistency of the four Sit and Reach measurements (right and left leg, pre- and post-test) was high, with a Cronbach’s alpha of 0.878, indicating strong reliability for assessing flexibility in this age group.

#### 2.4.2. Motor Coordination

The motor coordination test (3JS) has as its main objective the evaluation of the level of motor coordination of children between 6 and 11 years old. It consists of the development of seven consecutive tests: vertical jumps, turns, throws, kicks, running, bouncing, and driving in slalom [33] that bring about the five variables analyzed using this instrument: (i) motor coordination (MC), that is calculated by adding the scores obtained in each test of: jump, turn, throw, kick, run, bounce, and driving (between 7 and 28 points); (ii) locomotor coordination (LC), calculated by adding the jumping, turning, and running skills (between 3 and 12); (iii) object control coordination (OCC), obtained by adding the throwing, hitting, bouncing, and driving skills (takes variables between 4 and 16); (iv) the coordination of object control with the foot (FOC), which is calculated by adding the scores for hitting and driving (it takes variables from 2 to 8 points); and (v) the coordination of object control with the hand (HOC), which is obtained by adding the skills of throwing and dribbling. The procedure was implemented in accordance with the protocol outlined by Cenizo et al. [34]. In this study, the internal consistency of the 3JS subscales was excellent, with a Cronbach’s alpha of 0.901, confirming high reliability for this age group.

### 2.5. Intervention

Over a period of 12 weeks, students assigned to the experimental group took part in an integrated program that combined abacus-based cognitive activities with structured physical exercise. The program was delivered through two one-hour sessions per week and followed a three-phase format.

Each session began with a five-minute warm-up aimed at preparing the body for physical exercises through dynamic movement exercises. This initial phase also served to reinforce the correct manipulation of the abacus. Children practiced essential techniques, including using the thumb to slide beads upward, the index finger to move them downward, and exclusively using the index finger to handle the upper bead, which represents the value of five. Additionally, the warm-up included a brief revision of the mathematical operations introduced during the previous session.

The main phase, which lasted 45 min, integrated cognitive activities with physical exercises, allowing participants to learn how to use the abacus while developing motor skills. Regarding the abacus, a progressive method was followed. In the first session, the parts of the abacus and the value of each bead were explained. In sessions two and three, direct single-digit addition and subtraction were taught, allowing children to manipulate the beads without the need for formulas. In sessions four and five, the concept of mental calculations for direct addition and subtraction was introduced, requiring children to visualize the abacus in their mind and move the beads around mentally. Sessions six and seven addressed the concept of number “friends”, i.e., the numbers needed to reach another number; for example, to reach 5 from 1, you need 4. This concept was also used when there were not enough beads in a row to perform a direct calculation. In sessions eight and nine, “friends” were applied to subtraction situations when there were not enough beads available. In sessions ten and eleven, direct two-digit addition and subtraction was worked on, using two rows of the abacus. Finally, in the last three sessions, the children learned to perform direct addition and subtraction with three digits. Simultaneously, the physical exercises included 2–3 motor games specifically designed to complement the abacus-based learning, aiming to enhance physical abilities such as coordination, balance, and strength (Table 1).

These games were conducted in a playful and engaging setting that encouraged collaboration and teamwork among the children. Each physical exercise was intentionally linked to abacus practice, as students were asked to solve mathematical problems using the abacus after completing the motor tasks. This integrated strategy not only strengthened their physical competencies but also supported the connection between movement and mathematical learning, fostering a more holistic approach to their overall development.

In the correction phase, which lasted 10 min, the exercises carried out were reviewed, resolving the children’s doubts and consolidating the learning. During this time, group reflection was encouraged, where participants could share their experiences and achievements.

Finally, regular contact was maintained with the participants’ legal guardians to ensure that the activities carried out were exclusively those established in the program, ensuring the coherence of the intervention. This integrative model combined the best of cognitive learning and physical development, promoting meaningful and complete learning for the children.

### 2.6. Statistical Analysis

Descriptive statistics, including means and standard deviations, were calculated for all variables under investigation. To determine whether there were significant differences between groups, independent samples *t*-tests were employed. Prior to conducting these analyses, the assumptions of normality and homogeneity of variance were verified using the Kolmogorov–Smirnov and Levene’s tests, respectively. To evaluate the effects of the intervention, a mixed-design ANOVA was performed. In this analysis, the group variable (experimental vs. control) was treated as the between-subjects factor, while the time variable (pre- and post-intervention) served as the within-subjects factor. Additionally, to examine whether sex influenced the results, complementary analyzes of covariance (ANCOVA) were conducted using sex as a covariate. The primary outcome measures focused on coordination and flexibility, each assessed through appropriate evaluation tools. The analysis also considered potential interaction effects between group allocation and time of measurement. Effect sizes were calculated using Cohen’s d, with values interpreted as follows: < 0.2 for small effects, 0.5 for moderate effects, and > 0.8 for large effects. Statistical significance was set at *p* < 0.05. Observed power (1−β) values were also computed for all ANOVA tests using SPSS. For statistically significant effects, the power estimates exceeded the commonly accepted threshold of 0.80, indicating a low probability of Type II errors and supporting the robustness of the findings. All data were processed and analyzed using SPSS version 17.0.

## 3. Results

Participants had a mean age of 6.67 years, with a standard deviation of 1.02. Of the total sample, 52.40% were attending the first year of primary education, while 47.60% were enrolled in the second year. All students who took part in this study maintained an attendance rate exceeding 80%, which allowed them to complete the entire intervention program (Table 2).

### 3.1. Flexibility

In terms of right leg flexibility, participants in the CG reported higher values (1.37 ± 1.64) than those in the EG (1.29 ± 1.71) before the start of the intervention, but conversely, in the post-measure, the EG obtained higher results (2.12 ± 1.62 vs. 1.22 ± 1.74). The experimental group exhibited a statistically significant improvement from the pre- to the post-intervention assessment (t(40) = −3.043, *p* = 0.004), with a moderate effect size (Cohen’s d = 0.50). Post-intervention comparisons between groups also revealed significant differences favoring the experimental group (t(80) = −2.434, *p* = 0.017, Cohen’s d = 0.54). Additionally, an ANCOVA controlling for sex confirmed the significance of the group effect (F(1,79) = 10.481, *p* = 0.002, η^2^ = 0.116), with no significant effect of sex observed (*p* = 0.381). This indicates that the intervention’s effectiveness on right leg flexibility was independent of sex.

**Figure 2:**
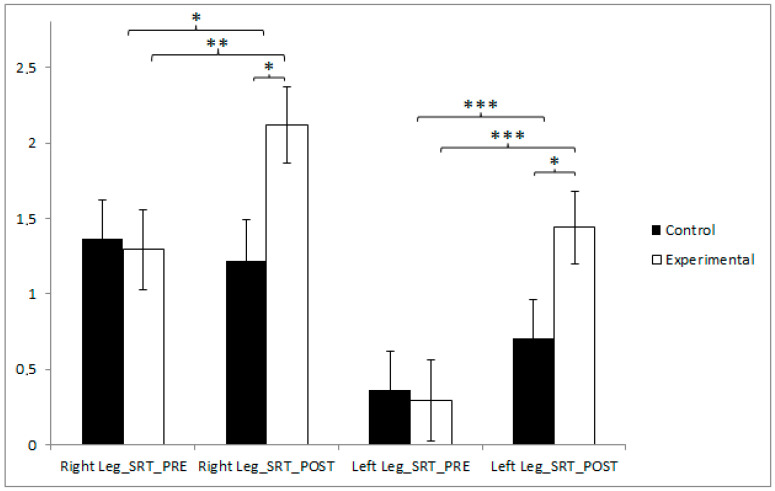
Inter- and intragroup comparisons regarding flexibility. * *p* < 0.05; ** *p* < 0.01; *** *p* < 0.001; SRT: Sit and Reach test.

A similar trend was observed for left leg flexibility. The participants in the CG reported higher values (0.37 ± 1.64) than those in the EG (0.29 ± 1.71) before the start of the intervention, but conversely, in the post-intervention measure, the EG obtained higher results (1.44 ± 1.53 vs. 0.71 ± 1.63). The training group recorded a significant enhancement between pre- and post-test measurements (t(40) = −4.150, *p* < 0.001), with a large effect size (Cohen’s d = 0.71). Moreover, intergroup comparison at the conclusion of the intervention indicated a statistically significant difference (t(80) = −2.092, *p* = 0.040), accompanied by a moderate effect size (Cohen’s d = 0.46), as shown in Figure 2 and Table 3. Additionally, an ANCOVA controlling for sex as a covariate confirmed the significance of the group effect (F(1,79) = 5. 467, *p* = 0.022, η^2^ = 0.064), while the sex covariate showed no significant influence (*p* = 0.723). These findings reinforce the robustness of the intervention’s effect on left leg flexibility.

### 3.2. Motor Coordination

**Figure 3:**
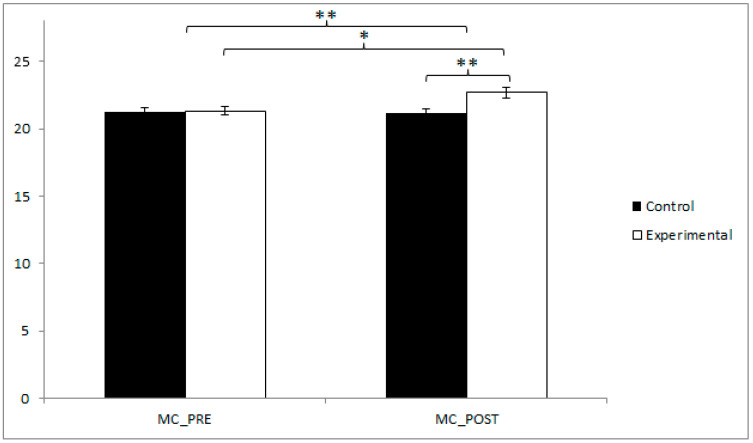
Inter- and intragroup comparisons regarding motor coordination. * *p* < 0.05; ** *p* < 0.01; MC: Motor Coordination.

In terms of motor coordination, the participants in the EG reported higher values (21.32 ± 1.81) than those in the CG (21.22 ± 1.88) before the start of the intervention, as well as in the post-intervention measure (22.68 ± 2.65 vs. 21.20 ± 1.66). The training group demonstrated statistically significant improvements from pre- to post-intervention (t(40) = −2.507, *p* = 0.016, Cohen’s d = 0.05). Furthermore, a significant difference was identified between the experimental and control groups at the post-intervention assessment (t(80) = −3.046, *p* = 0.003, Cohen’s d = 0.01). (Figure 3). Additionally, an ANCOVA controlling for sex confirmed the significance of the group effect (F(1,79) = 13.181, *p* = 0.000, η^2^ = 0.141), with no significant effect of sex observed (*p* = 0.473).

With respect to locomotor coordination, the participants in the EG reported higher values (9.61 ± 1.22) than those in the CG (9.51 ± 1.29) before the start of the intervention, as well as in the post-intervention measure (10.17 ± 1.24 vs. 9.34 ± 0.97). The training group exhibited statistically significant improvements from pre- to post-intervention (t(40) = −3.683, *p* = 0.001, Cohen’s d = 0.46). Moreover, significant differences were also identified between the experimental and control groups in the post-intervention assessment (t(80) = −5.232, *p* < 0.001, Cohen’s d = 0.75), indicating a moderate to large effect size. Additionally, an ANCOVA controlling for sex confirmed the significance of the group effect (F(1,79) = 9.351, *p* = 0.003, η^2^ = 0.105), with no significant effect of sex observed (*p* = 0.422).

**Figure 4:**
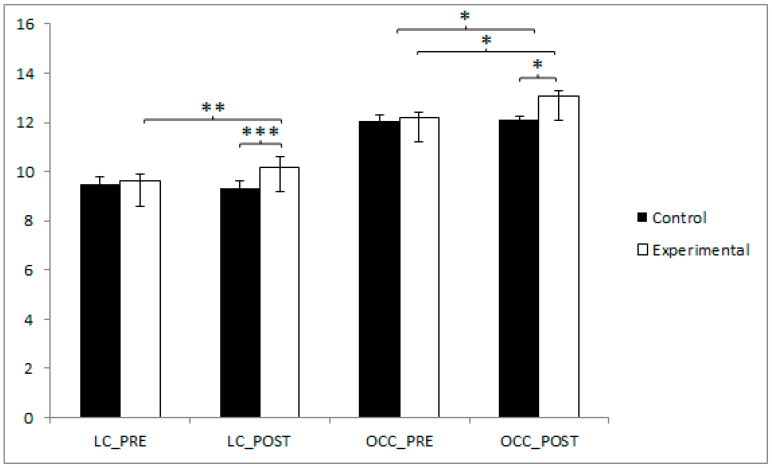
Inter- and intragroup comparisons regarding LC and OCC. * *p* < 0.05; ** *p* < 0.01; *** *p* < 0.001; LC: Locomotion Coordination; OCC: Object Control Coordination.

In terms of object control coordination, the participants in the EG reported higher values (12.20 ± 1.66) than those in the CG (12.07 ± 1.71) before the start of the intervention, as well as in the post-intervention measure (13.07 ± 2.22 vs. 12.10 ± 1.30). Pre–post comparisons within the training group revealed significant gains (t(40) = −2.677, *p* = 0.011, Cohen’s d = 0.44), and post-intervention results showed significant differences between groups (t(80) = −2.430, *p* = 0.017, Cohen’s d = 0.53). (Figure 4). Additionally, an ANCOVA controlling for sex confirmed the significance of the group effect (F(1,79) = 6.055, *p* = 0.016, η^2^ = 0.070), with no significant effect of sex observed (*p* = 0.725).

Regarding locomotor coordination, the participants in the CG reported higher values (5.20 ± 1.15) than those in the EG (5.17 ± 1.07) before the start of the intervention, but conversely, in the post-intervention measure, the EG obtained higher results (5.71 ± 1.40 vs. 5.12 ± 0.93). The training group demonstrated statistically significant improvements from pre- to post-intervention (t(40) = −3.683, *p* = 0.001, Cohen’s d = 0.46). Additionally, post-intervention comparisons between the experimental and control groups revealed statistically significant differences (t(80) = −5.232, *p* < 0.001, Cohen’s d = 0.75), reflecting a moderate to large effect size. Additionally, an ANCOVA controlling for sex confirmed the significance of the group effect (F(1,79) = 6.039, *p* = 0.016, η^2^ = 0.070), with no significant effect of sex observed (*p* = 0.180).

**Figure 5:**
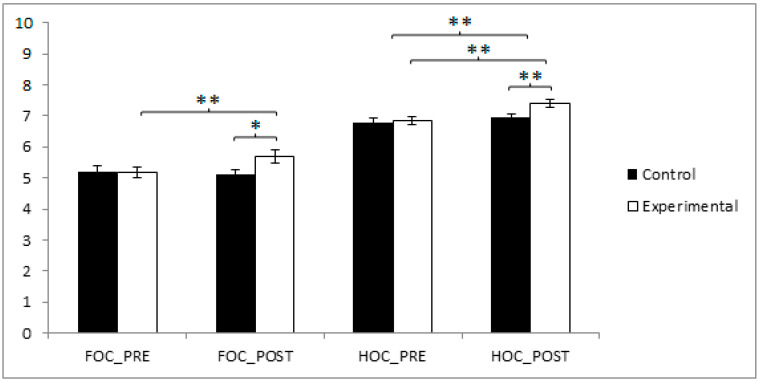
Inter- and intragroup comparisons regarding FOC and HOC. * *p* < 0.05; ** *p* < 0.01; FOC: Foot Object Coordination; HOC: Hand Object Coordination.

In the domain of object control coordination, the participants in the EG reported higher values (6.83 ± 0.83) than those in the CG (6.80 ± 0.81) before the start of the intervention, as well as in the post-intervention measure (7.41 ± 0.81 vs. 6.95 ± 0.67). The training group also showed significant improvements over time (t(40) = −2.677, *p* = 0.011, Cohen’s d = 0.44), and a significant difference was observed between groups at post-intervention (t(80) = −2.430, *p* = 0.017, Cohen’s d = 0.53) (Figure 5) (Table 2). Additionally, an ANCOVA controlling for sex confirmed the significance of the group effect (F(1,79) = 4.101, *p* = 0.046, η^2^ = 0.049), with no significant effect of sex observed (*p* = 0.328).

## 4. Discussion

The results of this study demonstrate that the combination of structured physical exercises and the use of the abacus in primary school children produce significant improvements in motor coordination and flexibility. The results of this study demonstrate that the combination of structured physical exercises and the use of the abacus in primary school children produces significant improvements in motor coordination and flexibility. These findings are consistent with previous studies that emphasize the importance of early physical exercise interventions in promoting motor skill acquisition, particularly coordination and flexibility [8,24,30]. Moreover, the observed improvements align with research highlighting the cognitive and motor stimulation benefits of combined physical-cognitive approaches [14,15]. While the results reinforce existing literature, it is important to interpret them with caution given the limitations of this study, especially the relatively short intervention period and the lack of long-term follow-up. These factors may influence the sustainability and generalizability of the improvements observed, and should be considered when applying these findings to other educational settings. From a pedagogical standpoint, the intervention offers practical insights into the integration of physical and cognitive tools within the classroom. The inclusion of abacus-based tasks linked to physical games may serve not only to enhance motor coordination, but also to reinforce concentration, attention, and engagement in children. Educators could adapt these tools during physical education or math sessions to foster holistic development. Such integration supports current educational paradigms that emphasize active learning and the value of embodied cognition in early education. This approach may also be particularly effective for students with developmental delays or learning difficulties, by providing alternative pathways for learning through movement.

Several studies support the idea that regular physical exercise is a key component of children’s motor development, improving skills such as coordination, balance, and agility [14,15,16,17,18,19,20,21,22,23,24,25,26,28,29,30,31,32,33,34,35,36]. Physical exercises, especially those that are structured and focus on specific motor skills, have been shown to be effective in strengthening both gross and fine motor skills [37,38]. In our study, children in the experimental group participated in games and physical exercises that stimulated skills such as coordination and flexibility, which is consistent with the literature that maintains that physical exercise programs at early ages are essential for the comprehensive development of these capacities [39,40].

Furthermore, recent research has explored how specific skill training can impact other areas of motor development. For example, Veldman et al. [41] and Scott et al. [42] found that physical exercise programs focused on coordination and balance can lead to improvements in agility and hand–eye coordination skills, which reinforces the results of our study. Likewise, Tomporowski et al. [14] argue that physical exercise improves executive control in children, which could help explain the observed improvements in precision and coordination in the experimental group of our study.

The use of the abacus in the educational and motor context is a less common approach, but our results suggest that its inclusion can enhance the development of fine motor skills and visual–motor coordination in children [28]. Previous studies have indicated that the practice of the abacus improves cognitive and fine motor skills, by requiring precise and repetitive movements with the fingers [42,43]. Such movements can help develop strength and precision in the muscles of the hands and fingers, which are fundamental for fine motor skills, as well as for hand–eye coordination, a key skill in general motor development [44,45]. In this sense, our findings extend this line of research by demonstrating that the abacus, in addition to its cognitive potential, can also be useful to improve motor skills when used in combination with physical exercises.

The improvement in flexibility observed in the experimental group is also in line with previous studies suggesting that physical exercises, especially those involving dynamic stretching and broad movements, promote the development of this skill at an early age [34,46]. Indeed, research has shown that flexibility is a fundamental physical skill that can significantly benefit from regular and structured physical exercise interventions [47]. The inclusion of stretching and broad movements in our intervention seems to have had a positive effect on this aspect, which is consistent with existing literature [48].

Our findings also provide a new perspective on the use of the abacus in the context of motor development. While several investigations have demonstrated the cognitive benefits of using the abacus, in particular in improving mathematical and mental calculation skills [49], few studies have explored its effects on physical and motor development. By combining the use of the abacus with physical exercises, our results suggest that the abacus may offer additional benefits in terms of coordination and fine motor skills. This supports the current trend in education that advocates active learning and movement in the classroom, which has also been proposed as a way to improve cognitive and academic performance in children [15].

However, some studies have obtained mixed results when combining physical and cognitive activities in educational contexts. Diamond et al. [15] emphasize that, while physical exercise can have benefits on self-control and attention, its effectiveness depends on factors such as the structure and duration of the interventions, as well as the individual characteristics of the children. In our study, the structure of the intervention, with a clear division into warm-up, main phase, and correction, seems to have facilitated motor learning and practice. However, future research could evaluate the effectiveness of this combination of tools in different populations and cultural contexts.

The results of this study have significant practical implications for the design of early childhood development programs. The integration of tools such as the abacus and structured physical exercise provides a comprehensive approach that encompasses both motor and cognitive development. This supports the growing consensus that educational programs should not focus exclusively on cognitive development, but also on the physical and social well-being of students [50]. From a practical perspective, the inclusion of tools such as the abacus and the structuring of physical exercise sessions within the classroom can be particularly useful to improve fine and gross motor skills in children at early stages of development. Specifically, physical education programs in the classroom could benefit from the incorporation of activities and tools that promote both motor and cognitive learning, providing a more holistic learning environment [51]. Furthermore, the combination of cognitive activities with physical exercises seems to be a promising strategy to improve attention.

An important limitation of this study is the sample size, which, although adequate to observe significant improvements, limits the generalizability of the results to a broader population. Future studies could consider larger and more diverse samples to examine whether the observed effects are maintained in different contexts. In addition, the duration of the intervention (12 weeks) allowed for the assessment of short-term improvements; however, long-term effects and their possible impact on other areas of cognitive and motor development were not observed. Longitudinal research could offer a more complete picture of the sustained benefits of this combination of activities. Another limitation to consider is the possible influence of external factors, such as motivation and family support on children’s activities. Although our study attempted to control these factors through contact with tutors, it is difficult to completely rule out their impact on the results. Future research could incorporate tools to measure motivation and family support to more accurately assess these factors.

## 5. Conclusions

In conclusion, the results of this study support the effectiveness of a multidimensional approach that integrates physical and cognitive activities, such as abacus-based exercises, in the development of coordination and flexibility in young children. This integrated method provides a practical and low-cost strategy that can be easily implemented by educators within regular classroom and physical education settings. Teachers can adapt similar activities to promote not only motor development but also attention, concentration, and engagement in learning tasks. Although further studies are needed to replicate these results in diverse populations and educational contexts, as well as to evaluate long-term effects, the findings offer promising insights for the design of holistic early childhood education programs. Future research may explore how this combined approach impacts additional cognitive and socio-emotional domains and how it can be adapted for use with children with different developmental needs.

## Figures and Tables

**Table 1:** Description of motor games.

Physical Abilities	Motor Games	Description
Coordination	Crazy traffic light	Children must start, stop, or change direction depending on the color announced by the teacher, enhancing visual–motor coordination and reaction speed.
	Obstacle courses	A sequence of movements through cones and jumping over hoops designed to develop agility and body control.
	Number relay race	Children take turns running to a board where they must place the correct number to complete a basic math operation, combining speed with mental calculation.
Balance	Acrobat path	Participants walk slowly and carefully along lines or ropes placed on the floor, focusing on dynamic balance and stability.
	Mathematical statues	Upon hearing a number, children freeze in a specific pose and remain still, promoting postural control and focused attention.
	Number jump	Based on the result of an abacus operation, children jump from one numbered floor mark to another in sequence
Strength	Cooperative pull	Children work in teams using elastic bands or ropes to create resistance, encouraging cooperative effort and upper body strength.
	Push and solve	In pairs, children press their palms against each other gently while mentally solving simple arithmetic problems.
	Mathematical load	Children carry light sandbags to a target, where they solve a math problem aloud before returning, integrating physical effort with cognitive processing.

**Table 2:** Participant characteristics prior to the intervention.

		Total (n = 82)	Experimental (n = 41)	Control (n = 41)	*p*-Value
**Age (years)**		6.67 ± 1.02	6.68 ± 1.04	6.66 ± 1.01	0.848
**Sex**	**Male**	48 (58.50)	23 (47.90)	25 (52.10)	0.394
	**Female**	34 (41.50)	18 (52.90)	16 (47.90)	
**School year**	**First course**	39 (47.60)	19 (46.30)	20 (48.80)	0.697
	**Second course**	43 (52.40)	22 (53.70)	21 (51.20)	
**Weight (kg)**		22.50 ± 4.23	22.63 ± 4.12	22.37 ± 4.38	0.705
**Height (m)**		1.20 ± 0.78	1.20 ± 0.07	1.21 ± 0.08	0.582
**BMI (kg/m^2^)**		22.50 ± 4.23	22.63 ± 4.12	22.37 ± 4.38	0.705
**Waist circumference (cm)**		57.11 ± 4.06	57.24 ± 4.12	56.98 ± 4.05	0.987
**Hip circumference (cm)**		61.48 ± 4.34	61.54 ± 4.40	61.41 ± 4.34	0.896
**Waist-to-hip ratio (cm)**		0.93 ± 0.01	0.93 ± 0.11	0.92 ± 0.01	0.963
**Right Leg_SRT**		1.33 ± 1.66	1.29 ± 1.71	1.37 ± 1.64	0.702
**Left Leg_SRT**		0.34 ± 1.67	0.29 ± 1.70	0.36 ± 1.63	0.702
**MC_3JS**		21.27 ± 1.83	21.32 ± 1.81	21.22 ± 1.88	0.811
**LC_3JS**		9.56 ± 1.25	9.61 ± 1.22	9.51 ± 1.29	0.789
**OCC_3JS**		12.13 ± 1.68	12.20 ± 1.66	12.07 ± 1.71	0.789
**FOC_3JS**		5.18 ± 1.10	5.17 ± 1.07	5.20 ± 1.15	0.626
**HOC_3JS**		6.82 ± 0.82	6.83 ± 0.83	6.80 ± 0.81	0.803

Values are expressed as mean ± standard deviation for continuous variables and as n (%) for categorical variables. BMI: Body Mass Index. SRT: Sit and Reach test. 3JS = Jump, Turn, and Slalom Test. MC: Motor Coordination. LC: Locomotor Coordination. OCC: Object Control Coordination FOC: Foot Object Coordination. HOC: Hand Object Coordination. cm: centimeters. kg: kilograms. m: meters.

**Table 3:** Effects of the intervention on flexibility and motor coordination.

	EG (n = 41)		CG (n = 41)		Group			Time			Group x Time		
	Pre	Post	Pre	Post	F(79)	*p*-Value	η^2^	F(79)	*p*-Value	η^2^	F(79)	*p*-Value	η^2^
**Right Leg_SRT**	1.29 ± 1.71	2.12 ± 1.62	1.37 ± 1.64	1.22 ± 1.74	1.504	*0.224*	0.018	5.136	*0.026*	0.060	10.481	*0.002*	0.116
**Left Leg_SRT**	0.29 ± 1.70	1.44 ± 1.53	0.36 ± 1.63	0.71 ± 1.64	1.086	*0.300*	0.013	18.680	*0.000*	0.189	5.467	*0.022*	0.064
**MC_3JS**	21.32 ± 1.81	21.22 ± 1.88	21.22 ± 1.88	21.20 ± 1.66	3.796	*0.055*	0.045	12.272	*0.001*	0.133	13.181	*0.000*	0.141
**LC_3JS**	9.61 ± 1.22	10.17 ± 1.24	9.51 ± 1.29	9.34 ± 0.97	3.956	*0.050*	0.047	2.660	*0.107*	0.032	9.351	*0.003*	0.105
**OCC_3JS**	12.20 ± 1.66	13.07 ± 2.22	12.07 ± 1.71	12.10 ± 1.30	2.514	*0.117*	0.030	6.767	*0.011*	0.078	6.055	*0.016*	0.070
**FOC_3JS**	5.17 ± 1.07	5.71 ± 1.40	5.20 ± 1.15	5.12 ± 0.93	1.606	*0.209*	0.020	3.488	*0.065*	0.042	6.039	*0.016*	0.070
**HOC_3JS**	6.83 ± 0.83	7.41 ± 0.81	6.80 ± 0.81	6.95 ± 0.67	3.276	*0.074*	0.039	11.392	*0.001*	0.125	4.101	*0.046*	0.049

Quantitative variables are presented as mean and standard deviation. EG: Experimental Group. CG: Control Group. SRT: Sit and Reach test. MC: Motor Coordination. LC: Locomotor Coordination; OCC: Object Control Coordination. FOC: Foot Object Coordination. HOC: Hand Object Coordination.

## Data Availability

The data are contained within the article.
